# Monoclinic polymorph of 2,5-bis[4-(dimethyl­amino)­styr­yl]-3,6-dimethyl­pyrazine

**DOI:** 10.1107/S1600536811006234

**Published:** 2011-03-12

**Authors:** Janina Fischer, Volker Schmitt, Dieter Schollmeyer, Heiner Detert

**Affiliations:** a University Mainz, Duesbergweg 10-14, 55099 Mainz, Germany

## Abstract

The title compound, C_26_H_30_N_4_, was prepared by condensation of tetra­methyl­pyrazine and dimethyl­amino­benzaldehyde and crystallizes from chloro­form/methanol in two different forms. Block-shaped crystals belong to the monoclinic crystal system and plates to the triclinic system. The two crystal forms differ in the arrangement of the centrosymmetric mol­ecules, which have nearly identical geometries. In the monoclinic crystals reported here, planar mol­ecules [maximum deviation = 0.062 (2) Å], with a *transoid* arrangement of the (*E*)-styryl units and completely planarized dimethylamino groups [sum of the C—N bond angles = 359.9 (2)°], form layers connected *via* H–π-stacking. The dihedral angle between the central and pendant rings is 1.30 (8)°. The triclinic polymorph contains two half molecules, both completed by crystallographic inversion symmetry.

## Related literature

The title compound was synthesized as a fundamental chromophore in a larger project focusing on solvatochromic and acidochromic dyes for sensing applications *via* one and two-photon excited fluorescence, see: Nemkovich *et al.* (2010 ▶); Schmitt *et al.* (2008 ▶); Detert & Schmitt (2006 ▶); Strehmel *et al.* (2003 ▶). Starting with 2,5-dimethyl­pyrazine, linear distyryl­pyrazines had been prepared by acid-catalyzed condensations with benzaldehyde (Takahashi & Satake, 1952 ▶) as well as *via* Siegrist reaction with the anils of alk­oxy­benzaldehydes (Zerban, 1991 ▶). Crystal data for the triclinic form have been deposited (CCDC 807782).
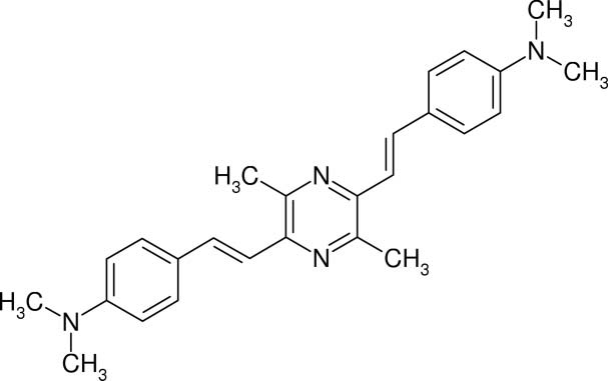


## Experimental

### 

#### Crystal data

C_26_H_30_N_4_
*M* *_r_* = 398.54
Monoclinic, 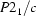

*a* = 6.0635 (5) Å
*b* = 15.5187 (13) Å
*c* = 12.8009 (12) Å
β = 113.449 (6)°
*V* = 1105.06 (17) Å^3^
*Z* = 2
Mo *K*α radiation
μ = 0.07 mm^−1^
*T* = 193 K
0.49 × 0.45 × 0.27 mm

#### Data collection

Bruker SMART CCD diffractometer
13517 measured reflections
2637 independent reflections
1711 reflections with *I* > 2σ(*I*)
*R*_int_ = 0.057

#### Refinement

*R*[*F* ^2^ > 2σ(*F* ^2^)] = 0.056
*wR*(*F* ^2^) = 0.170
*S* = 1.02
2637 reflections
139 parameters
H-atom parameters constrained
Δρ_max_ = 0.27 e Å^−3^
Δρ_min_ = −0.22 e Å^−3^

### 

Data collection: *SMART* (Bruker, 2006 ▶); cell refinement: *SAINT* (Bruker, 2006 ▶); data reduction: *SAINT*; program(s) used to solve structure: *SIR97* (Altomare *et al.*, 1999 ▶); program(s) used to refine structure: *SHELXL97* (Sheldrick, 2008 ▶); molecular graphics: *PLATON* (Spek, 2009 ▶); software used to prepare material for publication: *PLATON*.

## Supplementary Material

Crystal structure: contains datablocks I, global. DOI: 10.1107/S1600536811006234/si2330sup1.cif

Structure factors: contains datablocks I. DOI: 10.1107/S1600536811006234/si2330Isup2.hkl

Additional supplementary materials:  crystallographic information; 3D view; checkCIF report

## Figures and Tables

**Fig. 1.**
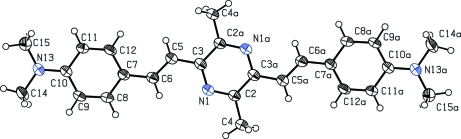
View of compound I. Displacement ellipsoids are drawn at the 50% probability level. Inversion-related atoms [1 - x, 1 - y, 1 - z] are shown with suffix a.

**Fig. 2.**
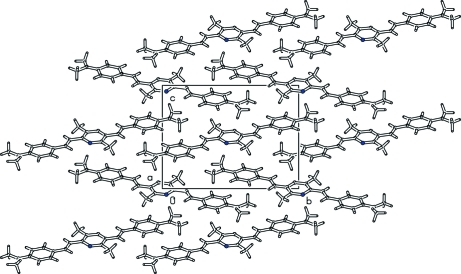
A packing section of the monoclinic crystal form viewed down the a axis.

**Fig. 3.**
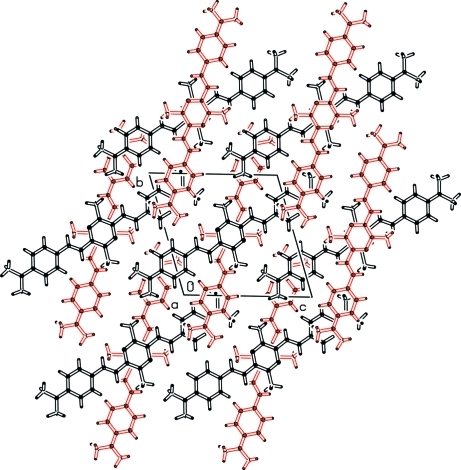
A packing section of the triclinic crystal form viewed down the a axis.

## supplementary crystallographic information

### Comment

The title compound (Fig1.) is formed *via* base-catalyzed condensation of *p*-dimethylaminobenzaldehyde and tetramethylpyrazine. Monoclinic and triclinic crystals are obtained by crystallization from chloroform/methanol.

The monoclinic crystal is built from parallel layers (Fig. 2) with a distance of the mean planes of 3.5 Å indicating a π-π-interaction of neighbouring molecules. Perpendicular to the pyrazine-N are the nitrogen atoms of the dimethylamino groups of molecules in the lower and upper layer. Each molecule is connected to six neighbouring molecules *via* H-π-interactions with distances H–centroid of the π-system in the range of 2.27 - 2.88 Å. These CH-π-bonds connect pyrazin-methyl groups C4—H with anilines, and dimethylamino groups C14—H with pyrazines and C15—H with aniline rings. The molecules are planar, C14 shows the largest deviation (0.062 (2) A) from the plane defined by all 15 non H-atoms. The bond length C10—N13 of only 1.368 (2) Å is close to the bond lengths in the central heterocycle (N1—C2: 1.333 (2) Å; N1—C3: 1.353 (2) Å) indicating a strong electronic interaction of terminal donors and the central pyrazine acceptor.

The triclinic form contains two independent half-molecules which both are completed by inversion symmetry [1 - *x*, 1 - *y*, 1 - *z* and -*x*, 1 - *y*, 1 - *z*], drawn with different colours in the packing diagram of Fig. 3.

These molecules are arranged in layers with a distance of 3.7 Å and a tilt angle of 4 °. The centroids of the pyrazine rings of layers A and B are collinear but the molecules are twisted about about 60 °. The layers are connected *via* hydrogen bonds from C14—H (A) to the aniline ring (B). Crystal data are deposited under CCDC 807782.

### Experimental

The title compound was prepared by adding potassium *tert*-butylate (1.50 g, 16.1 mmol) to a solution of tetramethylpyrazine (0.90 g, 6.70 mmol) and 4-dimethylaminobenzaldehyde (2.00 g, 13.41 mmol) in anhydrous DMF (25 ml). The mixture was stirred at 273 K under nitrogen until the aldehyde has been consumed (TLC). The mixture was diluted with water (75 ml) and the product extracted with chloroform, the solution dried with Na_2_SO_4_ and after evaporation of the solvent, the residue recrystallized from chloroform/methanol (1:1) to yield a mixture of block- and plate-shaped, dark red crystals. Yield: 1.42 g (54%), m.p.=522 K.

### Refinement

Hydrogen atoms attached to carbons were placed at calculated positions with C—H = 0.95 Å (aromatic) or 0.98–0.99 Å (*sp*^3^ C-atom). All H atoms were refined in the riding-model approximation with isotropic displacement parameters (set at 1.2–1.5 times of the *U*_eq_ of the parent atom).

### Crystal data

**Table:**

C_26_H_30_N_4_	*F*(000) = 428
*M**_r_* = 398.54	*D*_x_ = 1.198 Mg m^−^^3^
Monoclinic, *P*2_1_/*c*	Melting point: 522 K
Hall symbol: -P 2ybc	Mo *K*α radiation, λ = 0.71073 Å
*a* = 6.0635 (5) Å	Cell parameters from 3217 reflections
*b* = 15.5187 (13) Å	θ = 2.6–27°
*c* = 12.8009 (12) Å	µ = 0.07 mm^−^^1^
β = 113.449 (6)°	*T* = 193 K
*V* = 1105.06 (17) Å^3^	Block, orange
*Z* = 2	0.49 × 0.45 × 0.27 mm

### Data collection

**Table:**

Bruker SMART CCD diffractometer	1711 reflections with *I* > 2σ(*I*)
Radiation source: sealed Tube	*R*_int_ = 0.057
graphite	θ_max_ = 28.0°, θ_min_ = 2.2°
CCD scan	*h* = −7→7
13517 measured reflections	*k* = −20→19
2637 independent reflections	*l* = −16→16

### Refinement

**Table:**

Refinement on *F*^2^	Primary atom site location: structure-invariant direct methods
Least-squares matrix: full	Secondary atom site location: difference Fourier map
*R*[*F*^2^ > 2σ(*F*^2^)] = 0.056	Hydrogen site location: inferred from neighbouring sites
*wR*(*F*^2^) = 0.170	H-atom parameters constrained
*S* = 1.02	*w* = 1/[σ^2^(*F*_o_^2^) + (0.093*P*)^2^ + 0.1486*P*] where *P* = (*F*_o_^2^ + 2*F*_c_^2^)/3
2637 reflections	(Δ/σ)_max_ = 0.001
139 parameters	Δρ_max_ = 0.27 e Å^−^^3^
0 restraints	Δρ_min_ = −0.22 e Å^−^^3^

### Special details

**Table:**

Geometry. All e.s.d.'s (except the e.s.d. in the dihedral angle between two l.s. planes) are estimated using the full covariance matrix. The cell e.s.d.'s are taken into account individually in the estimation of e.s.d.'s in distances, angles and torsion angles; correlations between e.s.d.'s in cell parameters are only used when they are defined by crystal symmetry. An approximate (isotropic) treatment of cell e.s.d.'s is used for estimating e.s.d.'s involving l.s. planes.
Refinement. Refinement of *F*^2^ against ALL reflections. The weighted *R*-factor *wR* and goodness of fit *S* are based on *F*^2^, conventional *R*-factors *R* are based on *F*, with *F* set to zero for negative *F*^2^. The threshold expression of *F*^2^ > σ(*F*^2^) is used only for calculating *R*-factors(gt) *etc*. and is not relevant to the choice of reflections for refinement. *R*-factors based on *F*^2^ are statistically about twice as large as those based on *F*, and *R*- factors based on ALL data will be even larger.

### Fractional atomic coordinates and isotropic or equivalent isotropic displacement parameters (Å^2^)

**Table:**

	*x*	*y*	*z*	*U*_iso_*/*U*_eq_	
N1	0.3645 (2)	0.52967 (9)	0.55807 (11)	0.0361 (4)	
C2	0.3898 (3)	0.44536 (10)	0.54641 (13)	0.0340 (4)	
C3	0.4723 (3)	0.58565 (10)	0.51230 (13)	0.0347 (4)	
C4	0.2626 (3)	0.38669 (11)	0.59782 (16)	0.0437 (5)	
H4A	0.3808	0.3592	0.6661	0.066*	
H4B	0.1750	0.3423	0.5424	0.066*	
H4C	0.1492	0.4203	0.6185	0.066*	
C5	0.4434 (3)	0.67746 (11)	0.52817 (15)	0.0374 (4)	
H5	0.5138	0.7174	0.4943	0.045*	
C6	0.3227 (3)	0.70847 (11)	0.58799 (14)	0.0363 (4)	
H6	0.2543	0.6666	0.6203	0.044*	
C7	0.2832 (3)	0.79779 (11)	0.60981 (14)	0.0347 (4)	
C8	0.1390 (3)	0.81714 (11)	0.66978 (16)	0.0424 (5)	
H8	0.0713	0.7710	0.6959	0.051*	
C9	0.0920 (3)	0.90040 (11)	0.69218 (15)	0.0424 (5)	
H9	−0.0074	0.9101	0.7327	0.051*	
C10	0.1880 (3)	0.97122 (11)	0.65634 (13)	0.0352 (4)	
C11	0.3336 (3)	0.95248 (11)	0.59606 (14)	0.0374 (4)	
H11	0.4019	0.9985	0.5700	0.045*	
C12	0.3784 (3)	0.86880 (11)	0.57435 (14)	0.0383 (4)	
H12	0.4776	0.8589	0.5338	0.046*	
N13	0.1460 (3)	1.05436 (9)	0.67883 (13)	0.0443 (4)	
C14	−0.0132 (4)	1.07191 (13)	0.73601 (18)	0.0524 (5)	
H14A	−0.1665	1.0421	0.6962	0.079*	
H14B	−0.0415	1.1341	0.7357	0.079*	
H14C	0.0610	1.0513	0.8148	0.079*	
C15	0.2474 (3)	1.12637 (12)	0.64153 (16)	0.0479 (5)	
H15A	0.4228	1.1207	0.6724	0.072*	
H15B	0.2044	1.1802	0.6688	0.072*	
H15C	0.1838	1.1270	0.5581	0.072*	

### Atomic displacement parameters (Å^2^)

**Table:**

	*U*^11^	*U*^22^	*U*^33^	*U*^12^	*U*^13^	*U*^23^
N1	0.0347 (8)	0.0396 (8)	0.0399 (8)	0.0014 (6)	0.0210 (6)	−0.0010 (6)
C2	0.0300 (8)	0.0397 (9)	0.0351 (9)	0.0006 (6)	0.0161 (7)	−0.0006 (7)
C3	0.0304 (8)	0.0415 (10)	0.0342 (9)	0.0024 (6)	0.0151 (7)	−0.0013 (7)
C4	0.0462 (10)	0.0433 (10)	0.0542 (11)	0.0003 (8)	0.0334 (9)	−0.0002 (8)
C5	0.0374 (9)	0.0388 (9)	0.0416 (10)	0.0012 (7)	0.0215 (8)	−0.0008 (7)
C6	0.0342 (9)	0.0401 (9)	0.0378 (9)	−0.0004 (7)	0.0176 (8)	−0.0006 (7)
C7	0.0340 (9)	0.0390 (9)	0.0359 (9)	0.0022 (6)	0.0191 (7)	−0.0006 (7)
C8	0.0469 (10)	0.0433 (10)	0.0506 (11)	−0.0020 (7)	0.0338 (9)	0.0012 (8)
C9	0.0449 (10)	0.0476 (11)	0.0493 (11)	0.0020 (7)	0.0343 (9)	−0.0006 (8)
C10	0.0337 (9)	0.0408 (10)	0.0350 (9)	0.0029 (7)	0.0179 (7)	−0.0015 (7)
C11	0.0388 (9)	0.0398 (9)	0.0418 (10)	−0.0027 (7)	0.0249 (8)	−0.0008 (7)
C12	0.0363 (9)	0.0459 (10)	0.0424 (10)	0.0004 (7)	0.0258 (8)	−0.0024 (8)
N13	0.0517 (9)	0.0399 (9)	0.0532 (9)	0.0051 (6)	0.0336 (8)	−0.0023 (7)
C14	0.0531 (12)	0.0521 (11)	0.0653 (13)	0.0063 (9)	0.0376 (11)	−0.0115 (10)
C15	0.0501 (11)	0.0409 (11)	0.0577 (12)	−0.0017 (8)	0.0268 (10)	−0.0050 (8)

### Geometric parameters (Å, °)

**Table:**

N1—C2	1.333 (2)	C8—H8	0.9500
N1—C3	1.353 (2)	C9—C10	1.403 (2)
C2—C3^i^	1.413 (2)	C9—H9	0.9500
C2—C4	1.504 (2)	C10—N13	1.368 (2)
C3—C2^i^	1.413 (2)	C10—C11	1.415 (2)
C3—C5	1.459 (2)	C11—C12	1.377 (2)
C4—H4A	0.9800	C11—H11	0.9500
C4—H4B	0.9800	C12—H12	0.9500
C4—H4C	0.9800	N13—C15	1.445 (2)
C5—C6	1.341 (2)	N13—C14	1.451 (2)
C5—H5	0.9500	C14—H14A	0.9800
C6—C7	1.453 (2)	C14—H14B	0.9800
C6—H6	0.9500	C14—H14C	0.9800
C7—C12	1.401 (2)	C15—H15A	0.9800
C7—C8	1.406 (2)	C15—H15B	0.9800
C8—C9	1.378 (2)	C15—H15C	0.9800
			
C2—N1—C3	119.01 (14)	C8—C9—H9	119.4
N1—C2—C3^i^	120.87 (15)	C10—C9—H9	119.4
N1—C2—C4	116.32 (14)	N13—C10—C9	122.27 (15)
C3^i^—C2—C4	122.81 (15)	N13—C10—C11	121.15 (15)
N1—C3—C2^i^	120.12 (15)	C9—C10—C11	116.58 (15)
N1—C3—C5	117.47 (14)	C12—C11—C10	121.31 (15)
C2^i^—C3—C5	122.40 (15)	C12—C11—H11	119.3
C2—C4—H4A	109.5	C10—C11—H11	119.3
C2—C4—H4B	109.5	C11—C12—C7	122.44 (16)
H4A—C4—H4B	109.5	C11—C12—H12	118.8
C2—C4—H4C	109.5	C7—C12—H12	118.8
H4A—C4—H4C	109.5	C10—N13—C15	121.39 (14)
H4B—C4—H4C	109.5	C10—N13—C14	120.01 (15)
C6—C5—C3	123.56 (16)	C15—N13—C14	118.53 (14)
C6—C5—H5	118.2	N13—C14—H14A	109.5
C3—C5—H5	118.2	N13—C14—H14B	109.5
C5—C6—C7	128.43 (16)	H14A—C14—H14B	109.5
C5—C6—H6	115.8	N13—C14—H14C	109.5
C7—C6—H6	115.8	H14A—C14—H14C	109.5
C12—C7—C8	115.78 (15)	H14B—C14—H14C	109.5
C12—C7—C6	124.56 (15)	N13—C15—H15A	109.5
C8—C7—C6	119.66 (15)	N13—C15—H15B	109.5
C9—C8—C7	122.61 (15)	H15A—C15—H15B	109.5
C9—C8—H8	118.7	N13—C15—H15C	109.5
C7—C8—H8	118.7	H15A—C15—H15C	109.5
C8—C9—C10	121.28 (15)	H15B—C15—H15C	109.5
			
C3—N1—C2—C3^i^	0.3 (3)	C8—C9—C10—N13	−179.19 (17)
C3—N1—C2—C4	−179.17 (14)	C8—C9—C10—C11	0.3 (3)
C2—N1—C3—C2^i^	−0.3 (3)	N13—C10—C11—C12	179.28 (15)
C2—N1—C3—C5	−179.15 (14)	C9—C10—C11—C12	−0.2 (2)
N1—C3—C5—C6	2.0 (3)	C10—C11—C12—C7	0.2 (3)
C2^i^—C3—C5—C6	−176.81 (17)	C8—C7—C12—C11	−0.3 (3)
C3—C5—C6—C7	179.74 (15)	C6—C7—C12—C11	179.28 (15)
C5—C6—C7—C12	−3.1 (3)	C9—C10—N13—C15	179.76 (16)
C5—C6—C7—C8	176.42 (18)	C11—C10—N13—C15	0.3 (3)
C12—C7—C8—C9	0.4 (3)	C9—C10—N13—C14	−3.5 (3)
C6—C7—C8—C9	−179.23 (16)	C11—C10—N13—C14	177.07 (16)
C7—C8—C9—C10	−0.4 (3)		

Symmetry codes: (i) −*x*+1, −*y*+1, −*z*+1.
